# Brain structure differences among male schizophrenic patients with history of serious violent acts: an MRI voxel-based morphometric study

**DOI:** 10.1186/s12888-017-1263-9

**Published:** 2017-03-21

**Authors:** Noriomi Kuroki, Hiroko Kashiwagi, Miho Ota, Masanori Ishikawa, Hiroshi Kunugi, Noriko Sato, Naotsugu Hirabayashi, Toshio Ota

**Affiliations:** 1 Department of Psychiatry, National Center Hospital, National Center of Neurology and Psychiatry, 4-1-1 Ogawa-Higashi, Kodaira, Tokyo, 187-8551 Japan; 2 Department of Psychiatry, Saitama Medical University, 38 Morohongo Moroyama-machi, Iruma-gun, Saitama, 350-0495 Japan; 3 Department of Psychiatry, Tokyo Metropolitan Matsuzawa Hospital, 2-1-1 Kamikitazawa, Setagaya-ku, Tokyo, 156-0057 Japan; 4 Department of Mental Disorder Research, National Institute of Neuroscience, National Center of Neurology and Psychiatry, 4-1-1 Ogawa-Higashi, Kodaira, Tokyo, 187-8502 Japan; 5 Department of Social Welfare Services, Faculty of Human Science, Mejiro University, 4-31-1 Nakaochiai, Shinjuku-ku, Tokyo, 161-8539 Japan; 6 Department of Radiology, National Center Hospital, National Center of Neurology and Psychiatry, 4-1-1 Ogawa-Higashi, Kodaira, Tokyo, 187-8551 Japan

**Keywords:** Schizophrenia, MRI voxel-based morphometry, Violent acts, Premeditation

## Abstract

**Background:**

The biological underpinnings of serious violent behaviors in patients with schizophrenia remain unclear. The aim of this study was to identify the characteristics of brain morphometry in patients with schizophrenia and a history of serious violent acts, who were being treated under relatively new legislation for offenders with mental illness in Japan where their relevant action should be strongly associated with their mental illness. We also investigated whether morphometric changes would depend on types of serious violent actions or not.

**Methods:**

Thirty-four male patients with schizophrenia who were hospitalized after committing serious violent acts were compared with 23 male outpatients or inpatients with schizophrenia and no history of violent acts. T1-weighted magnetic resonance imaging (MRI) with voxel-based morphometry was used to assess gray matter volume. Additionally, patients with violent acts were divided based on whether their relevant actions were premeditated or not. The regional volumes of these two groups were compared to those of the control patient group.

**Results:**

Patients with schizophrenia and a history of serious violent acts showed significantly smaller regional volumes of the right inferior temporal area expanded to the middle temporal gyrus and the temporal pole, and the right insular area compared to patients without a history of violence. Patients with premeditated violent acts showed significantly smaller regional volumes of the right inferior temporal area, the right insular area, the left planum polare area including the insula, and the bilateral precuneus area including the posterior cingulate gyrus than those without a history of violence, whereas patients with impulsive violent acts showed significantly smaller volumes of only the right inferior temporal area compared to those without a history of violence.

**Conclusions:**

Patients with schizophrenia and a history of serious violent acts showed structural differences in some brain regions compared to those with schizophrenia and no history of violence. Abnormalities in the right inferior temporal area were relatively common but did not depend on whether the violent actions were premeditated or not, and abnormalities in a wider range may be attributed to not only planning the violent action against others but also to maintaining that plan.

**Trial registration:**

UMIN.ac.jp UMIN000008065. Registered 2012/05/31.

## Background

Although many individuals with schizophrenia do not show any antisocial behaviors, they hold a significant risk for violent acts compared to the general population [1]. Violent offenses are not necessarily among the general characteristics of schizophrenia, but a considerable number of patients with schizophrenia tend to repeat violent behaviors. The differences between patients with violent actions and those without remain unclear. Several factors have been suggested to distinguish the violent and non-violent groups, such as positive symptoms of schizophrenia [2], premorbid antisocial traits [3], and co-morbidity with substance disorders [1]. Genetic influences have also been proposed to have a significant impact on the risk of violence in schizophrenia [4]. It is suggested that biological and psychosocial factors may be intricately intertwined for forming a violent trait in schizophrenia.

The biological aspects of violent traits have been relatively well described among individuals with antisocial disorder and psychopathy, which are also strongly associated with violent traits. Historically, a violent trait has been thought to be associated with frontal lobe dysfunction. Some patients with organic frontal damage show similar behaviors to psychopathy [5], and frontal abnormalities have been reported in patients with antisocial personality disorder or psychopathy [6]. Morphological abnormalities in other regions such as the temporal pole [7–9], anterior cingulate cortex [10], nucleus accumbens [11], amygdala [12], and hippocampus [9, 13] were also reported in individuals with psychopathy or antisocial personality disorder. However, to what extent these abnormalities are shared among individuals with schizophrenia and violent traits remains unclear.

A few studies have examined the relationships between structural abnormalities and violent traits in patients with schizophrenia. Concerning the relationships between schizophrenia and aggression, several findings were reported, such as relationships between orbitofrontal cortex volumes [14], caudate volumes [15], and aggression using the Overt Aggression Scale in treatment-resistant patients with schizophrenia or schizoaffective disorder, as well as the correlation between cortical thinning in ventral prefrontal regions, urgency scores and the Aggression Questionnaire total score in patients with schizophrenia or schizoaffective disorder [16]. Several studies have included patients with a history of serious violence in addition to schizophrenia and/or antisocial personality disorder, and these studies reported volumetric abnormalities in brain regions including the medial temporal lobe. For patients with schizophrenia and a history of serious violence, Barkataki et al. reported reduced whole brain and increased putamen sizes compared with normal controls and non-violent patients with schizophrenia as well as reduced hippocampal volumes compared with normal controls and the antisocial personality disorder group [17]. Kumari et al. found elevated impulsiveness scores in participants with schizophrenia and a history of serious violence compared to non-violent patients and normal controls as well as a negative correlation between these factors and orbitofrontal gray matter volume and hippocampal volume in participants with schizophrenia and a history of violence [18]. Reduced hippocampal and parahippocampal volume among murderers with schizophrenia relative to non-murderers with schizophrenia was also reported by another group [19]. Volumetric abnormalities in other brain areas have also been reported, such as reduced thalamic volume in participants with schizophrenia or antisocial personality disorder and a history of psychosocial deprivation [20], as well as lower anterior cingulate volume in subjects with antisocial personality and/or schizophrenia and a history of serious violence, but not in patients with non-violent schizophrenia relative to normal subjects [10]. Regarding cortical thickness, Narayan et al. showed that violence was associated with cortical thinning in the medial inferior frontal and lateral sensory motor cortex, as only violent subjects with antisocial personality disorder exhibited cortical thinning in inferior mesial frontal cortices [21]. However, the study settings (i.e., community setting, or forensic ward-based) in these studies have varied, as did the groups compared (i.e., non-violent schizophrenia, antisocial personality disorder, and normal controls), and types of serious violent actions have not been well studied. Thus, whether specific structural abnormalities are related to violent traits in individuals with schizophrenia remains unknown.

A background of violent behaviors in schizophrenia consists of several factors. For example, murder based on delusion of persecution (i.e., goal-directed behavior) and physical attack during psychomotor excitement (i.e., impulsive behavior) may have different backgrounds, both on a psychological and a biological level. Thus, the results of studies of violent behaviors in individuals with schizophrenia may depend on the definition of the violent behaviors or the sampling of subjects, particularly subjects from a forensic setting vs. a community setting.

We examined hospitalized subjects with schizophrenia who had demonstrated serious physically violent behaviors, such as murder, attempted murder, and serious injury in community life, and were being treated under the Medical Treatment and Supervision Act (MTSA; also known as the Act on Medical Care and Treatment for Persons Who Have Caused Serious Cases Under the Condition of Insanity) [22]. This is relatively new legislation for offenders with mental disorders in Japan, which aims to promote the rehabilitation of persons who committed serious harm to others (homicide, robbery, bodily injury, arson, or a sex crime) while in a state of insanity or diminished responsibility and who are considered to be responsive to psychiatric treatment [23]. This new legislation should apply to subjects whose relevant action has been strongly associated with their mental illness. Therefore, we were able to sample subjects whose relationship between their relevant action and mental illness had been well examined, and investigate the association of types of their violent actions with morphometric characteristics. We analyzed high- resolution magnetic resonance imaging (MRI) findings with automated data processing, using voxel-based morphometry to detect regions associated with serious violent behaviors in patients with schizophrenia compared to non-violent patients with schizophrenia. We hypothesized that regional volume changes would depend on the types of serious violent actions, and we performed subgroup analysis by dividing patients with a history of serious violence into two groups according to whether their relevant actions were premeditated or not. The regional volumes of these two groups were compared to those of the control patient group.

## Methods

### Subjects

Thirty-four male patients with schizophrenia who had a history of seriously violent behaviors against others (the forensic group) were recruited from inpatients at the forensic unit of the National Center of Neurology and Psychiatry Hospital, Tokyo, Japan under the MTSA. Twenty-three age-matched male control patients with schizophrenia who had no history of violent behaviors (the control patient group) were recruited among inpatients and outpatients (10:13, respectively) at the National Center of Neurology and Psychiatry Hospital. Because there were few female patients with schizophrenia and a history of serious violent acts, female subjects were excluded from this study.

Inclusion criteria for both groups were as follows: (1) ages 20 to 60 years; (2) right-handed; (3) a diagnosis of schizophrenia according to the Diagnostic and Statistical Manual of Mental Disorders, Fourth Edition, Text Revision [24]. Exclusion criteria for both group were as follows: (4) a diagnosis of mental retardation; (5) a history of seizure; (6) a history of head trauma with loss of consciousness; (7) a history of neurological disorder. Forensic patients met the following criteria: (1) hospitalized under the MTSA; (2) had committed murder, attempted murder, or serious injury to others (relevant action) in a state of insanity or diminished responsibility; (3) were capable of participating in the study as determined through an evaluation by the multidisciplinary treatment team. Control patients met the following criteria: (1) had never been hospitalized for harming others; (2) had no history of physical violence (score of ≤1 on the Gunn and Robertson Scale for Violence [25]).

Data on the duration of education, history of substance abuse, age at onset and duration of illness, duration of medication, duration of untreated psychosis, and medication use at the time of MRI scan were available for all subjects. Ten forensic patients and no control patients had a history of substance abuse. One forensic patient and 5 control patients received no antipsychotic medication, although all other patients from both groups were receiving antipsychotic medication when the MRI scan was acquired. Two forensic patients and no control patients were taking clozapine. There were no antipsychotic medication-naive patients in either group. All medication dosages were converted to chlorpromazine equivalents [26]. Clinical symptoms of all patients were evaluated using the Positive and Negative Syndrome Scale (PANSS; [27, 28]) within 2 months of the date of MRI. The Wallwork/Fortgang five-factor model for PANSS was used for further analyses [29].

All forensic patients were hospitalized and had received psychiatric treatment for approximately 2 months prior to hospitalization under the MTSA to determine whether they should be treated under MTSA or not. The relationship between their relevant actions and mental disorder was evaluated in detail. Detailed clinical data were available for forensic patients during their hospitalization. The forensic group was divided into two groups for additional analysis, depending on whether they had planned their relevant action of violence for a certain period of time or not. Those who had premeditated their assaults prior to meeting with their victims on the day of their relevant actions or at least 1 hour before their actions were included in “the premeditated action group”; otherwise, the patients were included in "the impulsive group." The premeditated action group consisted of 15 subjects, including 4 patients with a history of substance abuse. The impulsive group consisted of 19 subjects, including 6 patients with a history of substance abuse.

Demographic data and clinical information are shown in Table 1.

**Table 1:** Demographic data and clinical Information

	Forensic group				Statistical analysis								
	Total ( *n* = 34)	Premeditated action group ( *n* = 15)	Impulsive group ( *n* = 19)	Control patient group ( *n* = 23)	Forensic ( *n* = 34) vs. Control ( *n* = 23) ^a^			Premeditated ( *n* = 15) vs. Control ^b^			Impulsive ( *n* = 19) vs. Control ^c^		
	Mean ± SD	Mean ± SD	Mean ± SD	Mean ± SD	t or U	df	p	t or U	df	p	t or U	df	p
Age, years (range)	40.6 ± 9.5 (22–58)	44.1 ± 9.2	37.9 ± 9.1	36.8 ± 11.0 (20–53)	1.385	55	0.172	2.112	36	0.042	0.338	40	0.737
Duration of education, years	12.4 ± 2.7	11.9 ± 2.2	12.9 ± 3.1	14.4 ± 2.0	2.965	55	0.004	3.673	36	0.001	1.939	40	0.060
Substance abuse, Alcohol/Others/Both	2/6/2	1/3/0	1/3/2	0/0/0									
Age of onset	25.4 ± 7.2	26.3 ± 8.5	24.7 ± 6.1	22.0 ± 6.7	1.834	55	0.072	1.743	36	0.090	1.398	40	0.170
Duration of illness, years	15.6 ± 10.0	18.0 ± 10.6	13.8 ± 9.3	15.0 ± 10.4	0.227	55	0.822	0.854	36	0.399	0.403	40	0.689
Duration of untreated psychosis, months	26.2 ± 41.8	36.2 ± 55.3	18.3 ± 26.2	18.8 ± 36.3	465		0.219	216		0.202	249		0.429
Duration of medication *a* , years	13.2 ± 10.2	14.4 ± 10.5	12.2 ± 10.1	13.6 ± 10.1	0.164	55	0.870	0.233	36	0.817	0.457	40	0.650
Medication dose, mg ^d^	706.9 ± 606.4	682.3 ± 543.7	726.3 ± 665.7	477.3 ± 434.8	476.5		0.164	212.5		0.235	264		0.249
Medication type, Typical/Atypical/Both/Non	2/18/13/1	1/5/8/1	1/13/5/1	2/12/4/5	7.000	3	0.072 ^f^	5.883	3	0.117 ^f^	7.041	3	0.071 ^f^
PANSS ^e^													
Positive	12.2 ± 4.8	14.3 ± 5.3	10.5 ± 3.8	9.4 ± 3.6	2.356	55	0.022	3.445	36	0.001	0.952	40	0.347
Negative	17.1 ± 7.0	19.7 ± 6.7	15.0 ± 6.8	14.7 ± 6.2	1.292	55	0.202	2.344	36	0.025	0.130	40	0.897
Disorganization	8.4 ± 3.2	9.2 ± 3.4	7.7 ± 2.9	6.8 ± 3.7	1.737	55	0.088	2.020	36	0.051	0.910	40	0.368
Depressive	6.7 ± 2.6	7.4 ± 2.9	6.1 ± 2.3	7.0 ± 3.2	0.420	55	0.676	0.395	36	0.695	1.024	40	0.312
Excitement	8.3 ± 3.5	8.4 ± 3.4	8.2 ± 3.8	6.7 ± 3.0	1.775	55	0.081	1.627	36	0.112	1.450	40	0.155
Intracranial volume, ml	1619.8 ± 154.2	1589.4 ± 173.6	1643.7 ± 137.1	1618.0 ± 136.8	0.045	55	0.964	0.565	36	0.576	0.606	40	0.548

^a^ Forensic group vs. control patient group

^b^ Premeditated action group vs control patient group

^c^ Impulsive group vs control patient group

^d^ Chlorpromazine equivalent

^e^ The Wallwork/Fortgang five-factor model for PANSS

^f^ Likelihood ratio test

This study was approved by the Ethical Review Board of the National Center of Neurology and Psychiatry. All participants were given a complete description of the study and provided written informed consent to participate.

### MRI acquisition and processing

Experiments were performed on a 3-tesla (3T) MRI system (Philips Medical Systems, Best, the Netherlands). High spatial resolution, 3-dimensional (3D) T1-weighted images were used for morphometric study. 3D T1-weighted images were acquired in the sagittal plane (repetition time (TR)/echo time (TE), 7.18/3.46 ms; flip angle, 10°; effective section thickness, 0.6 mm; slab thickness, 180 mm; matrix, 384 × 384; field of view (FOV), 261 × 261 mm; number of signals acquired, 1), yielding 300 contiguous slices through the brain.

Voxel-based morphometry analysis was performed using SPM12 (Statistical Parametric Mapping, Functional Imaging Laboratory (FIL), the Wellcome Trust Centre for NeuroImaging, Institute of Neurology at University College London (UCL), UK) under Matlab 7.14.0 (MathWorks, Natick, MA, USA). Structural T1-weighted MR images were segmented into gray matter, white matter, cerebrospinal fluid, bone, soft tissue, and air/background after bias regularization. Images of gray matter and white matter were spatially normalized to the Montreal Neurological Institute (MNI) space through Diffeomorphic Anatomic Registration using the Exponentiated Lie Algebra (DARTEL) algorithm [30, 31]. We modulated the images by the Jacobean determinants derived from the spatial normalization to MNI space using DARTEL to preserve volume information. Images were smoothed with an 8-mm, full width at half maximum Gaussian kernel.

### Statistical analysis

Independent samples *t*-tests or Mann-Whitney’s U tests were performed to assess differences between the forensic and the control groups in terms of age at scan, duration of education, age at onset, duration of illness, duration of medication, duration of untreated psychosis, medication dosage converted to chlorpromazine equivalents, composite scores for five factors of PANSS (e.g., positive symptoms, negative symptoms, disorganization/concrete symptoms, depressive symptoms, and excitement symptoms), and intracranial volumes (e.g., total gray matter volume (GMV) + total white matter volume + total cerebrospinal fluid volume; ICV). *T*-tests were performed when variables were normally distributed according to Shapiro-Wilk tests; otherwise, Mann-Whitney’s U tests were performed. The likelihood ratio test was performed for comparison of medication type (i.e., only typical/only atypical/both types/no medication) between two groups. Similarly, independent samples *t*-tests or Mann-Whitney’s U tests for these clinical variables were performed between the premeditated action group and the control patient group, as well as between the impulsive group and the control patient group.

We performed voxel-level comparison of GMV scaled by ICV between the forensic group and control group of patients with schizophrenia, using a two-sample *t*-test while considering other clinical factors when the significant group differences were identified as covariates. Group comparisons were assessed using the cluster-level family-wise error (FWE) correction for multiple comparisons, where the initial voxel threshold was set to uncorrected *p* = 0.001 and clusters were considered significant when falling below a cluster-corrected p(FWE) = 0.05. Prior to the comparison, we applied masking voxels in order to exclude non-brain voxels [32]. A history of substance abuse can affect brain morphometry, and previously shown to be associated with violence risk [1]. To eliminate the influence of substance abuse, a similar voxel-level comparison of GMV between the forensic group without patients with a history of substance abuse and the control patient group was performed, as no patients had a history of substance abuse in the control patient group.

We performed similar voxel-level comparison of GMV between the premeditated action group and the control patient group, as well as between the impulsive group and the control patient group.

## Results

No significant differences between the forensic group and the control patient group were identified in terms of age at scan, age at first episode, duration of illness, duration of untreated psychosis, duration of medications, and doses of antipsychotics. The forensic group showed a significantly lower duration of education compared to the control patient group. The forensic group also showed significantly higher composite score on positive symptoms compared to the control patient group. No significant differences were found for the other components of the PANSS. Duration of education and scores of positive symptoms were used as covariates for further voxel-level comparisons. No significant differences were found in ICV. Regarding comparisons between the premeditated action group and the control patient group, and between the impulsive group and the control patient group, the premeditated action group showed a significantly lower duration of education and higher scores of positive symptoms compared to the control patient group, while no significant differences were found in these variables between the impulsive group and the control patient group. There were also no significant differences in the other variables between the impulsive group and the control patient group. However, significant differences were found in terms of age at scan and scores of negative symptoms between the premeditated action group and the control patient group.

**Fig. 1:**
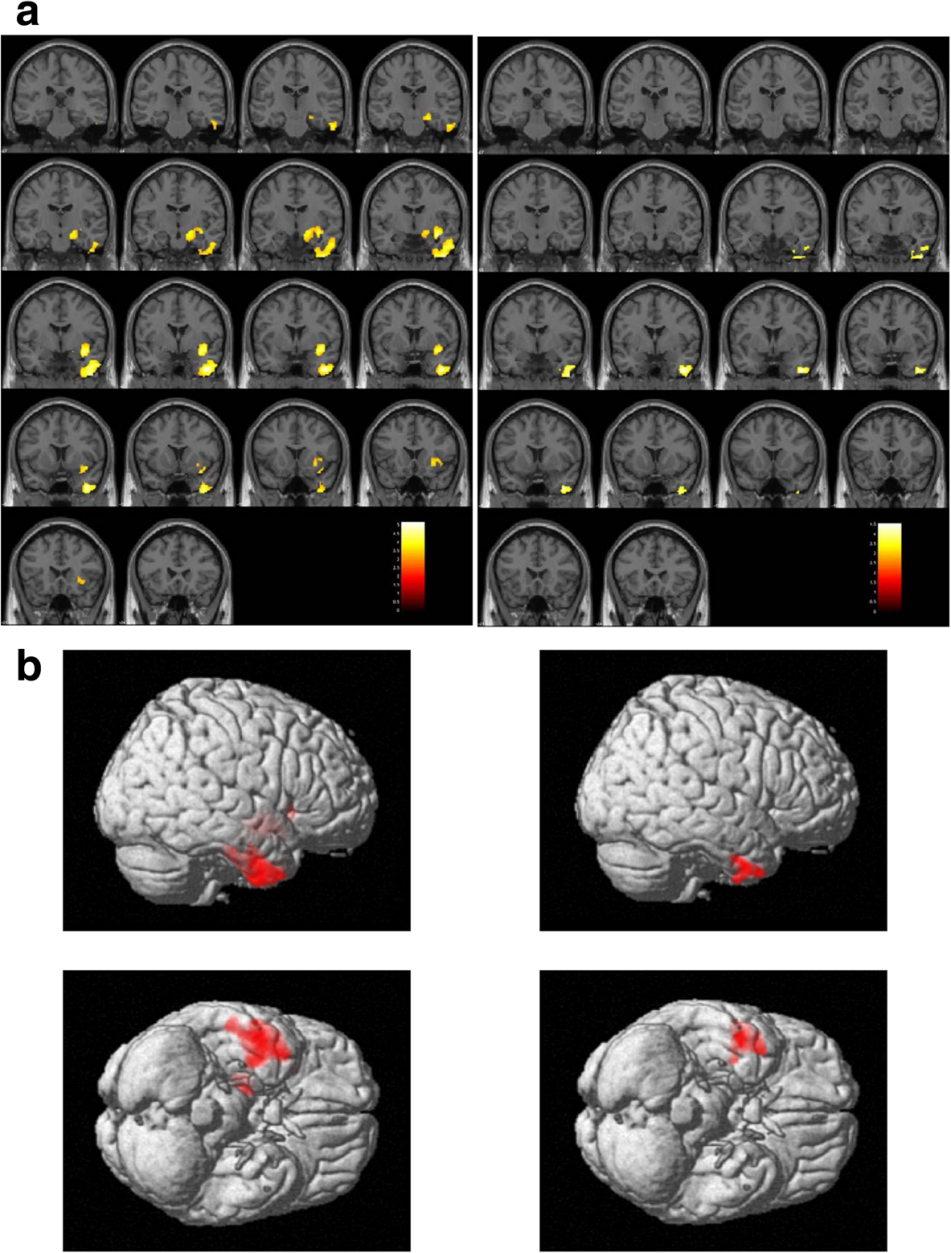
Brain regions with low gray matter volume in the forensic group. The initial voxel threshold: uncorrected *p* = 0.001, the cluster-level FWE correction, and a cluster-corrected p(FWE) < 0.05. *Left column* : Low gray matter volume in the forensic group ( *n* = 34) compared with the control patient group ( *n* = 23). Cluster For-1 (Table 2 ): The anterior part of the right inferior temporal gyrus expanded to the fusiform gyrus, the middle temporal gyrus, and the temporal pole. Cluster For-2 (Table 2 ): The right insula expanded to the ventral diencephalon. *Right column* : Low gray matter volume in the forensic group not including subjects with a history of substance abuse ( *n* = 24) compared with the control patient group ( *n* = 23). Cluster For(noS)-1 (Table 2 ): The anterior part of the right inferior temporal gyrus expanded to the temporal pole. **a** Montage of coronal slices y = −27 to 24 (MNI space). The maps were overlaid on the images of a single subject provided in SPM12. Right is right. **b** The maps rendered on the brain surface provided in SPM12

Statistical analysis showed that the forensic group had significantly lower GMV in the anterior part of the right inferior temporal gyrus expanded to the middle temporal gyrus, the temporal pole, and the fusiform gyrus (Table 2 cluster For-1, Fig. 1a, b left), as well as the right insula expanded to the ventral diencephalon (Table 2 cluster For-2, Fig. 1a left), compared to the control patient group. After excluding cases with a history of substance abuse, statistical significance remained for the right inferior temporal area expanded to the right temporal pole (Table 2 cluster For(noS)-1, Fig. 1a, b right).

**Table 2:** Gray matter volume reduction in patients with a history of serious violence relative to control patients

Cluster	Cluster-level *p* value (FWE-corrected)	Number of voxels	Area	Peak coordinate^a^		
				x	y	z
Forensic group (*n* = 34) vs. Control patient group (*n* = 23)						
For-1	0.001	3336	Right ITG, Right TP, Right MTG	44	0	-42
				46	-6	-50
				57	-6	-33
For-2	0.003	2369	Right PINS, Right AINS, Right VD	38	-6	-16
				40	3	-9
				20	-10	-10
Forensic group excluded subjects with a history of substance abuse (*n* = 24) vs. Control patient group (*n* = 23)						
For(noS)-1	0.043	1194	Right ITG, Right TP	45	0	-40
				48	-6	-50
				42	9	-42

*ITG* inferior temporal gyrus, *MTG* middle temporal gyrus, *TP* temporal pole, *AINS* anterior insula, *PINS* posterior insula, *VD* ventral diencephalon

^a^MNI space. Top 3 separate (>8 mm apart) maxima within a cluster

**Fig. 2:**
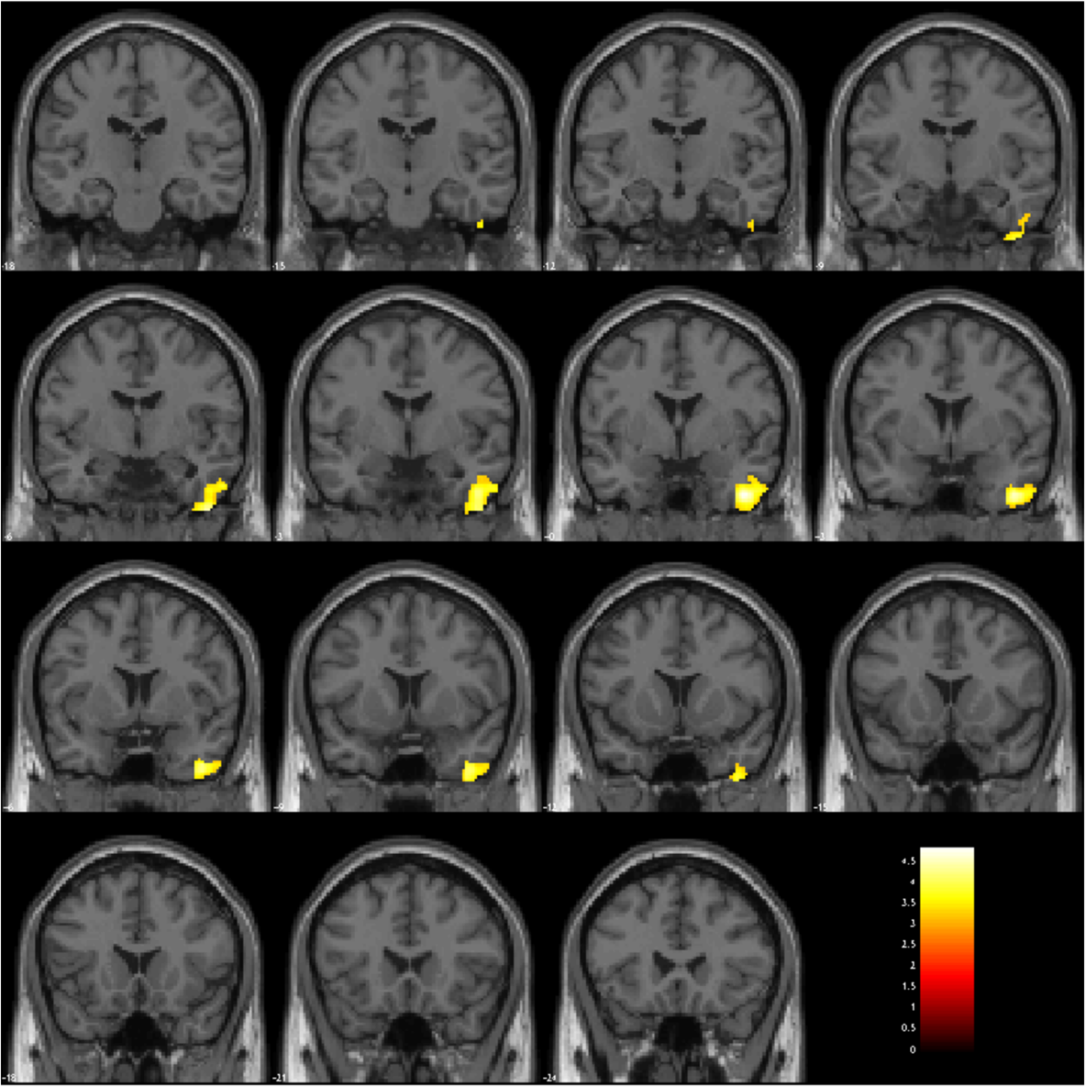
Brain regions with low gray matter volume in the premeditated action group. The initial voxel threshold: uncorrected *p* = 0.001, the cluster-level FWE correction, and a cluster-corrected p(FWE) < 0.05. Left: Montage of coronal slices y = −18 to 24 (MNI space). Right: Montage of sagittal slices x = −12 to 14 (MNI space). The maps were overlaid on the images of a single subject provided in SPM12. Cluster Prem-1 (Left, Table 3 ): The area including the right temporal pole, the inferior temporal gyrus, the fusiform gyrus, insula, and the ventral diencephalon. Cluster Prem-2 (Left, Table 3 ): The left planum polare and insula. Cluster Prem-3 (Right, Table 3 ): Bilateral precuneus and posterior cingulate gyrus

**Fig. 3:**
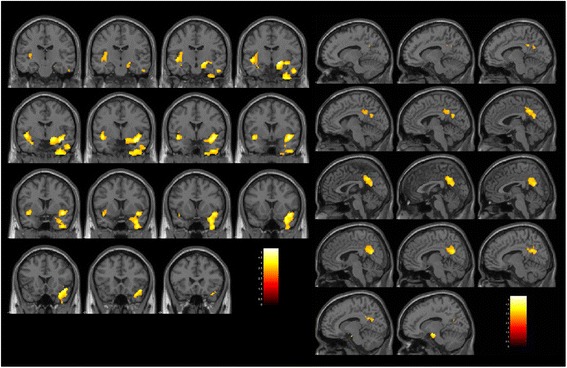
Brain regions of low gray matter volume in the impulsive group. The initial voxel threshold: uncorrected *p* = 0.001, the cluster-level FWE correction, and a cluster-corrected p(FWE) < 0.05. Montage of coronal slices y = −18 to 24 (MNI space). The maps were overlaid on the images of a single subject provided in SPM12 Cluster Imp-1 (Table 3): The right inferior temporal gyrus expanded to the temporal pole

The premeditated action group showed significantly lower GMV in the wide area including the right temporal pole, the inferior temporal gyrus, the fusiform gyrus, insula, the ventral diencephalon (Table 3 cluster Prem-1, Fig. 2 left), the left planum polare and insula (Table 3 cluster Prem-2, Fig. 2 left), and the bilateral precuneus and posterior cingulate gyrus (Table 3 cluster Prem-3, Fig. 2 right) compared to the control patient group. The impulsive group showed significantly lower GMV only in the right inferior temporal area expanded to the right temporal pole compared to the control patient group (Table 3 cluster Imp-1, Fig. 3).

**Table 3:** Gray matter volume redunction in the impulsive group and the premeditated action group, compared with the control group

Cluster	Cluster-level *p* value (FWE-corrected)	Number of voxels	Area	Peak coordinate^a^		
				x	y	z
Premeditated action group (*n* = 15) vs control group (*n* = 23)						
Prem-1	<0.001	6836	Right VD, Right AINS, Right PINS	20	-10	-10
				42	4	-9
				38	-4	-16
Prem-2	0.009	1789	Left PP, Left PINS, Left AINS	-45	-8	-4
				-42	-2	-9
				-33	-6	-22
Prem-3	0.005	2109	Left PCu, Right PCu, Left PCgG	0	-50	36
				6	-62	22
				-9	-44	28
Impulsive group (*n* = 19) vs control group (*n* = 23)						
Imp-1	0.02	1456	Right ITG, Right TP	45	0	-40
				46	-6	-50
				42	8	-44

*ITG* inferior temporal gyrus, *TP* temporal pole, *VD* ventral diencephalon, *AINS* anterior insula, *PINS* posterior insula, *PP* planum polare, *PCu* precuneus, *PCgG* posterior cingulate gyrus

^a^MNI space. Top 3 separate (>8 mm apart) maxima within a cluster

## Discussion

In this study, GMV was compared between patients with schizophrenia and a history of serious violence and patients with schizophrenia and no history of violent behaviors. Patients with a history of serious violence showed lower GMV in the right inferior temporal area (the anterior part of the right inferior temporal gyrus expanded to the fusiform gyrus, the middle temporal gyrus, and the temporal pole) and the right insular area (the right insula expanded to the ventral diencephalon) than those without a history of violence after controlling for lower years of education and higher positive symptom scores. Of note, the difference in the right inferior temporal area (the anterior part of the right inferior temporal gyrus and temporal pole) was not able to be explained by a history of substance abuse. GMV was also compared between three patient groups divided based on types of violent behaviors (i.e., patients with premeditated violent behaviors, patients with impulsive violent behaviors, and patients with schizophrenia and no history of violence). Both the premeditated action and impulsive group showed significantly lower GMV in the right inferior temporal area (the right inferior temporal gyrus expanded to the temporal pole) than the control patient group, whereas only the premeditated action group had significantly lower GMV in the right insular area (the right insula expanded to the ventral diencephalon), the left planum polare area (the left planum polare expanded to the insula), and the bilateral precuneus area (bilateral precuneus and posterior cingulate gyrus) than the control patient group.

Only a few studies have reported significant differences in regional volume among individuals with schizophrenia and a history of serious violent behaviors, particularly when compared to individuals with schizophrenia and no history of serious violence. The right inferior temporal area, including the temporal pole and the middle temporal gyrus, and the right insular area were newly reported in this study as associated with violent behavior. The right insular area was extended to the medial temporal area including the hippocampus as previously reported [17–19], but only in the premeditated action group. The forensic subjects in this study had committed serious harm to others while in a state of insanity or diminished responsibility. Due to the nature of this act, their relevant actions were strongly related to their psychiatric illness and were less related to an antisocial personality trait. Thus, the characteristics of the subjects in this study contribute to the available knowledge on violent behaviors in schizophrenia.

The inferior temporal gyrus is well known as the ventral stream of visual processing, referred to as the “what” stream, which processes the color and form of an object [33]. The ventral stream is suggested to work with the amygdala and other brain regions and contribute to the evaluation of biological significance of affective stimuli [34]. The fusiform gyrus is part of the ventral stream and is involved in face recognition [35]; this region is also suggested to be involved in the recognition of emotional stimuli, working together with the amygdala [36]. The temporal pole plays a role in top-down modulation of the ventral stream [37, 38] and is involved in emotional processing associated with stimuli of various modalities [39]. Reduced GMV in the right temporal pole and inferior temporal gyrus is shown in both the premeditated action and impulsive groups, indicating that the disturbance of integrated visual and other information processing and related emotional processing may be relatively common in individuals with schizophrenia and a history of serious violence.

The premeditated action group showed reduced GMV across a wide brain area including the insula compared with the impulsive group. This finding cannot be simply attributed to sample size. The premeditated action group had higher age at scan than the control patient group, but there were no differences in duration of illness. The premeditated action group showed higher score of negative symptoms as well as positive symptoms than the control patient group, whereas the impulsive group showed no significant differences in any clinical variables than the control patient group. More severe symptoms may be attributed to reduced GMV across a wide brain area. The insula is believed to play a role in recognition of the inner state of the body and is also involved in imagining, observing and executing an experience, such as another’s physical pain [40] or feelings of disgust [41]. Disturbance of this function may contribute to a lack of inhibition towards physically harming others. The precuneus is thought to be involved in the interwoven network of the neural correlates of self-consciousness, which is engaged in self-related mental representations during rest as well as other integrated tasks, including visuo-spatial imagery and episodic memory retrieval [42], and is also considered part of the “theory of mind (ToM)” network [43]. The function of recognizing others’ emotional pain may depend on the recognition of ToM. Lack of empathy based on the recognition of others’ physical and/or emotional pain may be attributed to a lack of inhibition for physically violent behaviors. More severe abnormalities in recognizing others’ pain may be attributed to not only planning the violent action towards others but also carrying out that plan.

It is interesting that the temporal pole and the insula, which are newly reported in this study regarding schizophrenia, were previously reported in individuals with psychopathy or antisocial behavioral problems, while our subjects were suggested to have less antisocial personality traits. One possibility is that a violent trait may depend on the same function both in individuals with antisocial problems and schizophrenia. There is also the possibility that an emotional processing disturbance in the temporal pole and the disturbance of recognizing another’s physical pain in the insula are common in violent traits both of schizophrenia and psychopathy/antisocial personality disorder. Another possibility is that subclinical psychopathic traits may have effects on morphometric changes even in our subjects with schizophrenia and a history of serious violence. Several reports have shown that individuals with schizophrenia and a history of violent offenses also possess psychopathic traits or antisocial traits [44, 45]. The biopsychosocial background of violence in people with schizophrenia may be heterogeneous. Morphometric findings regarding the relationship between schizophrenia and violence may depend on the type of violent behaviors performed by subjects with schizophrenia.

There are several limitations to this study. First, the subject population included men only. Second, several factors that may impact morphometric data were not available, such as IQ or socioeconomic status. There was a significant difference in the duration of education between groups, which may be reflected in these factors. Although we used the duration of education as a covariate, the influence of these cofounding factors may not have been fully eliminated. Third, long duration of illness and medication may affect our findings. Most subjects in this study had long duration of illness and were taking antipsychotic for a long time. We did not find significant differences in duration of illness, duration of untreated psychosis, and duration of medication. However, we could not fully eliminate the influence of long duration of illness and medication. Fourth, we could not fully eliminate the influence of substance abuse. Significant differences were found between the forensic group excluding subjects with substance abuse and the control patient group, but these differences were limited to the right inferior temporal area. We were unable to distinguish whether this finding was due to the sample size or not or whether a history of substance abuse was attributed to the other findings of our study. Fifth, we did not evaluate psychopathic traits, and we do not know to what extent psychopathic traits were associated with our findings. Moreover, classified the forensic patients based on how long they held the plan for their relevant violent actions; however, at the time of their relevant actions, impulsivity was likely attributed to these actions to a certain extent even in the premeditated action group. We also did not evaluate cognitive aspects in this study. Thus, we do not know the psychological functions associated with holding the plan aimed at harming others.

## Conclusions

Morphometric differences were identified between patients with schizophrenia with or without a history of serious violent acts, and such differences may depend on the type of violent actions committed. Abnormalities in the right inferior temporal gyrus expanded to the temporal pole were common but did not depend on whether the violent actions were premeditated or not, while abnormalities in a wider range of brain areas may be attributed to not only planning a violent action against others but also holding that plan. Patients with schizophrenia and a history of serious premeditated violent actions may have different risk for violent actions from those with other types of violent actions, and different treatment strategy may be needed for them. Morphometric measurement may be useful for assessment of violent patients in the future. Further studies are needed to determine which cognitive functions are related to these differences, and these findings may contribute to a more theoretical therapy for patients with schizophrenia and a history of serious violence.

## Abbreviations

GMV: Gray matter volume
ICV: Intracranial volume
MTSA: The Medical Treatment and Supervision Act, also known as the Act on Medical Care and Treatment for Persons Who Have Caused Serious Cases Under the Condition of Insanity
