# Erratum to: Brain structure differences among male schizophrenic patients with history of serious violent acts: an MRI voxel-based morphometric study

**DOI:** 10.1186/s12888-017-1302-6

**Published:** 2017-04-12

**Authors:** Noriomi Kuroki, Hiroko Kashiwagi, Miho Ota, Masanori Ishikawa, Hiroshi Kunugi, Noriko Sato, Naotsugu Hirabayashi, Toshio Ota

**Affiliations:** 1 Department of Psychiatry, National Center Hospital, National Center of Neurology and Psychiatry, 4-1-1 Ogawa-Higashi, Kodaira, Tokyo 187-8551 Japan; 2 Department of Psychiatry, Saitama Medical University, 38 Morohongo Moroyama-machi, Iruma-gun, Saitama 350-0495 Japan; 3 Department of Psychiatry, Tokyo Metropolitan Matsuzawa Hospital, 2-1-1 Kamikitazawa, Setagaya-ku, Tokyo, 156-0057 Japan; 4 Department of Mental Disorder Research, National Institute of Neuroscience, National Center of Neurology and Psychiatry, 4-1-1 Ogawa-Higashi, Kodaira, Tokyo 187-8502 Japan; 5 Department of Social Welfare Services, Faculty of Human Science, Mejiro University, 4-31-1 Nakaochiai, Shinjuku-ku, Tokyo, 161-8539 Japan; 6 Department of Radiology, National Center Hospital, National Center of Neurology and Psychiatry, 4-1-1 Ogawa-Higashi, Kodaira, Tokyo 187-8551 Japan

## Erratum

This article [1] has been updated to correct the image order. In the original article, Figs. 2 and 3 were interchanged. The figures are now in the correct position. This mistake was carried forward by production, and thus is not the fault of any authors of the manuscript.
